# Progressive pulmonary fibrosis in a murine model of Hermansky-Pudlak syndrome

**DOI:** 10.1186/s12931-022-02002-z

**Published:** 2022-05-04

**Authors:** Shachar Abudi-Sinreich, Steven P. Bodine, Tadafumi Yokoyama, Nathanial J. Tolman, Michal Tyrlik, Lauren C. Testa, Chen G. Han, Heidi M. Dorward, Stephen M. Wincovitch, Yair Anikster, William A. Gahl, Resat Cinar, Bernadette R. Gochuico, May Christine V. Malicdan

**Affiliations:** 1 Human Biochemical Genetics Section, National Human Genome Research Institute (NHGRI), National Institute of Health (NIH), Bethesda, MD 20892 USA; 2 The Sackler School of Medicine, Tel Aviv University, Tel Aviv, Israel; 3 UDP Translational Laboratory, NIH Undiagnosed Diseases Program, National Human Genome Research Institute (NHGRI), National Institutes of Health (NIH), Bethesda, MD 20892 USA; 4 National Human Genome Research Institute (NHGRI) Cytogenetics and Microscopy Core, National Institute of Health (NIH), Bethesda, MD 20892 USA; 5 Edmond and Lily Safra Children’s Hospital, Sheba Medical Center, Ramat Gan, Israel; 6 Laboratory of Physiologic Studies, National Institute on Alcohol Abuse and Alcoholism (NIAAA), National Institute of Health (NIH), Rockville, MD 20852 USA

**Keywords:** Pulmonary fibrosis, Rare disease, Interstitial lung disease, Translational disease models, Bleomycin

## Abstract

**Background:**

HPS-1 is a genetic type of Hermansky-Pudlak syndrome (HPS) with highly penetrant pulmonary fibrosis (HPSPF), a restrictive lung disease that is similar to idiopathic pulmonary fibrosis (IPF). Hps1^*ep/ep*^ (pale ear) is a naturally occurring HPS-1 mouse model that exhibits high sensitivity to bleomycin-induced pulmonary fibrosis (PF). Traditional methods of administering bleomycin as an intratracheal (IT) route to induce PF in this model often lead to severe acute lung injury and high mortality rates, complicating studies focusing on pathobiological mechanisms or exploration of therapeutic options for HPSPF.

**Methods:**

To develop a murine model of HPSPF that closely mimics the progression of human pulmonary fibrosis, we investigated the pulmonary effects of systemic delivery of bleomycin in Hps1^*ep/ep*^ mice using a subcutaneous minipump and compared results to oropharyngeal delivery of bleomycin.

**Results:**

Our study revealed that systemic delivery of bleomycin induced limited, acute inflammation that resolved. The distinct inflammatory phase preceded a slow, gradually progressive fibrogenesis that was shown to be both time-dependent and dose-dependent. The fibrosis phase exhibited characteristics that better resembles human disease with focal regions of fibrosis that were predominantly found in peribronchovascular areas and in subpleural regions; central lung areas contained relatively less fibrosis.

**Conclusion:**

This model provides a preclinical tool that will allow researchers to study the mechanism of pulmonary fibrosis in HPS and provide a platform for the development of therapeutics to treat HPSPF. This method can be applied on studies of IPF or other monogenic disorders that lead to pulmonary fibrosis.

**Supplementary Information:**

The online version contains supplementary material available at 10.1186/s12931-022-02002-z.

## Background

Hermansky-Pudlak syndrome (HPS) is an autosomal recessive disorder characterized by oculocutaneous albinism, a bleeding diathesis, inflammatory bowel disease and, in some genetic types, fatal pulmonary fibrosis (PF) [1]. To date, eleven types of HPS caused by biallelic mutations in eleven genes were reported, with HPS-1 being the the most prevalent [2]. Individuals with HPS-1, HPS-2 and HPS-4 develop HPS pulmonary fibrosis (HPSPF), a restrictive lung disease in which fibrosis occurs bilaterally and predominantly in the periphery of the lung [3, 4]. These disorders are characterized by irreversible and progressive fibrosis of the lung parenchyma and interalveolar septa. Very few drugs has been approved by the US Food and Drug Administration to treat progressive fibrosing interstitial lung disease, including Nintedanib [5], a tyrosinase-kinase inhibitor shown to inhibit processes involved in the progression of lung fibrosis. Nonetheless no specific treatment has been approved for HPSPF [6, 7]. Nintedanib might delay the progression of HPSPF but will not cure or reverse the disease but further studies are needed. Thus, there is an urgent need to generate in vivo models to clarify the mechannisms underlying disease and to test therapies.

HPSPF has been clinically compared to idiopathic pulmonary fibrosis (IPF), which occurs spontaneously in most patients. Amongst all genetic pulmonary fibrosis syndromes, HPSPF is the most penetrant and provides an excellent model for studying the cellular pathogenesis of PF [8]. HPSPF and IPF also share similarities in lung histopathology; both are characterized by irreversible and progressive fibrosis of the lung parenchyma and interalveolar septa, ultimately leading to respiratory failure. There are, however, features that are specific to HPSPF, including foamy alveolar macrophages and enlarged alveolar type II cells (AECII). IPF, on the other hand, manifests in patients mostly over age of 50, whereas HPSPF occurs as early as late adolescence (30 years of age) [9]. Moreover, the later onset of disease in IPF influences the survival rate in these two disorders; 50% of the IPF patients have 3 years survival rate whereas HPSPF patients live as long as 10 years after diagnosis [4]. Since HPSPF shares similar features with idiopathic pulmonary fibrosis, the identification of targets of therapy in this study can be used to understand a more commonly occurring but similarly fatal IPF.

HPS mouse models, including Hps1^*ep/ep*^ (pale ear) and Hps2^*pe/pe*^ (pearl), have facilitated research on HPS pulmonary fibrosis [10, 11]. Although Hps1^*ep/ep*^ or Hps2^*pe/pe*^ do not develop spontaneous pulmonary fibrosis, both HPS models display enhanced lung fibrosis in response to inhalation of fibrogenic stimuli when compared to similarly challenged wild type models [12–14]. Instillation of bleomycin into the lungs of rodents causes acute lung injury, inflammation, and subsequent interstitial fibrosis in rodents. Previous studies demonstrated that either oropharyngeal and intratracheal administration of bleomycin to HPS mouse models leads to an early and exaggerated inflammatory response, resulting in severe weight loss and high mortality [12, 15].

In this study, we aimed to develop a murine model of HPS pulmonary fibrosis that better resembles human disease and provides a new preclinical tool to further study mechanisms of disease, novel therapies for HPSPF and more common pulmonary fibrosis syndromes. We investigated bleomycin-induced pulmonary fibrosis in Hps1^*ep/ep*^ using a subcutaneous minipump to continuously infuse systemic bleomycin, but at a slower rate than acutely delivered intrtracheal or oroparyngeal route. Our hypothesis was that subcutaneous bleomycin challenge will improve mice survival and will lead to gradually progressive pulmonary fibrosis in Hps1^*ep/ep*^ that closely resembles that of human HPSPF.

## Materials and methods

### Mice

Breeding pairs of *Hps1* gene mutant (Hps1^*ep/ep*^) mice on the C57BL/6 J background were kindly provided by Dr. Susan H. Guttentag, University of Pennsylvania. C57BL/6 J mice were used as wild type (WT) controls. Procedures on Hps1^*ep/ep*^ mice were conducted on 8–12 weeks old male mice. Mice were bred in a pathogen-free barrier facility in accordance with NIH guidelines and approved by the NHGRI Animal Care and Use Committee (ACUC), under the protocol G-14-3 “Mouse models for disorders of lysosomes and lysosomal-related organelles”.

### Administration of bleomycin

Oropharyngeal administration of bleomycin, which allows direct aspiration of bleomycin, was performed by an expert scientist as previously described [16] with slight modifications to the dose based on the increased sensitivity of Hps1^*ep/ep*^ to bleomycin. Mice were placed on an adjustable stand and an external light source was directed over the trachea to visualize the vocal cords. Bleomycin (Hospira Inc.) was delivered at doses of 0.1 (n = 14), 0.2 (n = 14), 0.3 (n = 14), 0.5 (n = 13) or 1 U/Kg (n = 13). For control, mice were given saline (n = 6). Mice were followed for 14 days and their body weight was recorded; mice that lost 25% of baseline body weight were euthanized.

Systemic administration of bleomycin by subcutaneous osmotic minipump was performed as previously described [17]. Bleomycin was delivered continuously at 0 (n = 18), 15 (n = 5), 30 (n = 5), 45 (n = 13) or 60 (N = 26) U/Kg in 0.9% saline in the suprascapular subcutaneous space of Hps1^*ep/ep*^ mice via a surgically implanted 1007D Azlet osmotic minipumps on days 0 to 7. C57BL/6 J wild type were challenged with 0 (n = 6) or 100 U/Kg (n = 26). On day 7, minipumps were removed. Mice were euthanized at 0, 14, 21, 28, or 42 days post minipump implantation.

### Histopathology

Mice were euthanized using 100–150 μL of intraperitoneal 1.25% tribromoethanol unless specified otherwise. After euthanasia, the chest cavity was opened and all lobes of the right lung were inflated with 500 μL of 10% formalin intratracheally, removed, and immersed in formalin. Skin tissue taken from regions distant from the suprascapular area, kidney, and lung tissues were embedded in paraffin, sectioned at 5 µm, and stained with haematoxylin and eosin (H & E), and Masson’s trichrome. Bright field photomicrographs of stained tissues were imaged randomly without area selection using a Zeiss AxioObserver Z1 widefield microscope equipped with a plan-apochromat (N.A. 0.45) objective lens, a motorized scanning stage, and an Axiocam MRc5 color CCD camera (Zeiss).

### Hydroxyproline quantification

Prior to right lung inflation, the left main bronchus was ligated, and the left lung was excised, snap frozen in liquid nitrogen and stored at – 80 °C. Hydroxyproline quantification was performed via LC–MS/MS as previously described [16]. Assays were done in technical duplicates.

### Fluorescent in situ hybridization (FISH)

RNAscope® Multiplex Fluorescent Kit v2 (Advanced Cell Diagnostics (ACD), Newark, CA, USA) was used to perform RNA-probe based fluorescent in situ hybridization (FISH) on 5 µm thick formalin fixed paraffin embedded (FFPE) mouse lung tissue. Lung tissue sections were baked at 60 ºC for 60 min, deparaffinized in two 5 min washes of Hemo-De® (VWR, Randor, PA, USA) followed by two 5 min washes of 100% ethanol, and then air-dried at room temperature for 10 min. Endogenous peroxidases were quenched with a 10 min incubation with hydrogen peroxide and protease (RNAscope^®^, ACD, Cat. 322330) at room temperature, and sections were washed twice with water. Antigen retrieval was performed by steaming lung tissue at 99–100 ºC in water for 10 s, then 1X RNAscope^®^ Target Retrieval (ACD, Cat. 322000) for 15 min. Slides were then moved immediately to water for 15 s, to 100% ethanol for 3 min and then air dried. Hydrophobic barrier was drawn (PAP pen, Abcam, Cat. ab2601), then 5 drops of Protease Plus (ACD, Cat. 322331) were added to digest the lung tissue at 40º for 30 min; slides were then washed twice with water.

RNAscope® ISH probes from ACD were used at their recommended dilutions and incubated on lung tissue at 40 ºC for 120 min. The probes used from ACD were *TGF-β* in channel 1 (ACD, 322331), *Il1-β* in channel 2 (316891-C2), and *Polr2a* in channel 3 for control (312471-C3). The samples were washed twice in 1X RNAscope^®^ Wash Buffer (31009) and stored in 5X SSC buffer (Quality Biological, 351003101) overnight. Slides were washed twice with washing buffer and treated with Amp 1–3 (323100) according to the manufacturer’s instructions. The fluorophores (Perkin Elmer, Waltham, MA, USA) used to visualize the probes were fluorescein (NEL741E001KT), cyanine 3 (NEL744001KT), and cyanine 5 (NEL745E001KT), which were diluted 1:750 in a TSA buffer (ACD, 322810). Slides were treated with HRP-C1-3 to develop a signal, then fluorophore (C1-fluorescein, C2-Cyanine 3, C3-Cyanine 5) and HRP blocker (ACD, 323100) as specified in the manufacturer’s protocol. Slides were treated with DAPI for 30 s and mounted with VECTASHIELD^®^ Antifade Mounting Medium (Vector laboratories, Burlingame, CA, USA; Cat. H-1000-10) prior to applying a coverslip.

After 24 h, random fields in peripheral and central lung regions were imaged using a Zeiss 510 META confocal laser-scanning microscope (Carl Zeiss, Thornwood, NY, USA). All in situ experiments were performed for data collection in technical duplicates. Several optimization steps were condeucted before final data collection.

### Data analysis

The RNAScope data were analyzed using ZEN Blue software and the Image Analysis Wizard. The analysis parameters were set using a small subset of images and later validated on a larger set. The approximate accuracy was determined by visual inspection of the mask overlayed on the microscope image. The final analysis routine described in Additional file 3: Table S1 was saved into an Image Analysis Settings file. This file was loaded as a Settings parameter of the “Analyze Batch to File” processing option in ZEN. The analysis was performed in the Single function mode.

## Results

### Variability of pulmonary fibrosis in Hps1^***ep/ep***^ mice following oropharyngeal administration of bleomycin

**Fig. 1:**
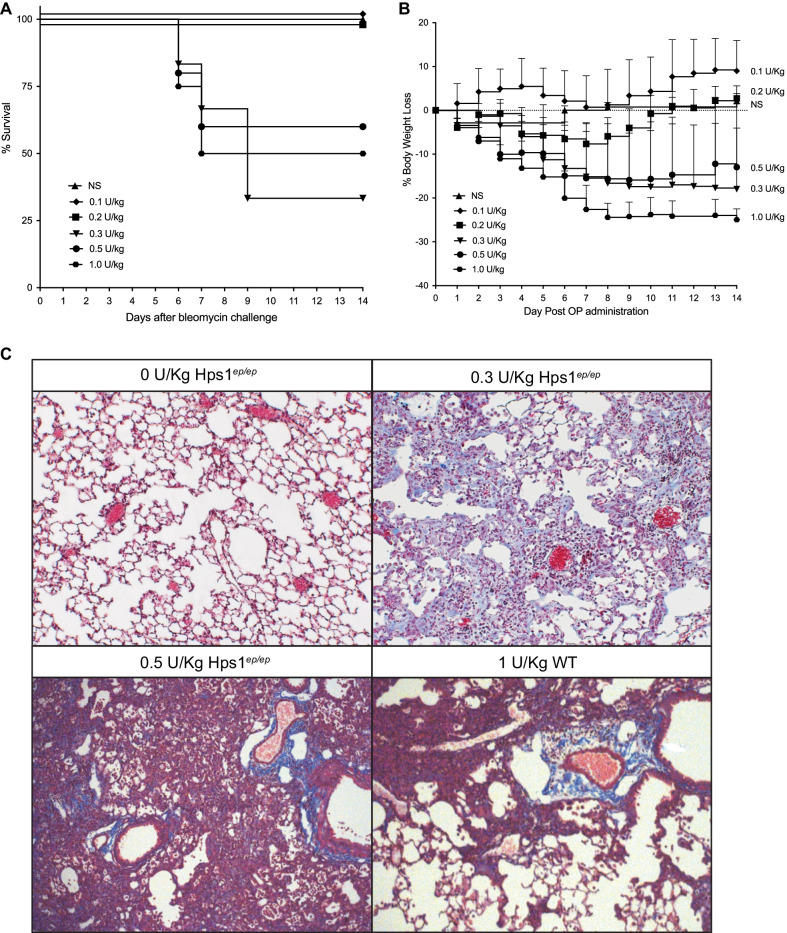
Variable severity of pulmonary fibrosis induced by oropharyngeal administration of bleomycin in Hps1^*ep/ep*^ mice or C57BL/6 J wild type mice. **A** Survival rates of Hps1^*ep/ep*^ mice with pulmonary fibrosis induced by oropharyngeal bleomycin (0.1–1 U/Kg) are variable and not dependent on bleomycin dose. **B** Percentage of body weight loss of Hps1^*ep/ep*^ mice on days 0 to 14 compared to baseline weight on day 0 (before bleomycin administration) is also variable. **C** Masson’s trichrome stained lung slides (20×) of Hps1^*ep/ep*^ mice treated with 0, 0.3 and 0.5 U/Kg compared to wild type C57BL/6J treated with 1 U/Kg bleomycin. An example of the variability in response to bleomycin administration which was not dose dependent: a dose of 0.3 U/Kg bleomycin caused some areas of fibrosis in addition to inflammation compared to higher doses of 0.5 U/Kg and 1 U/Kg that showed only major inflammation 14 days post the administration

Age- and weight-matched male Hps1^*ep/ep*^ mice were challenged utilizing oropharyngeal aspiration with normal saline or bleomycin at doses of 0.1 to 1.0 U/Kg. Mice were monitored for 14 days. Mortality in these mice ranged from 0 to 70% by 14 days post bleomycin administration (Fig. 1A). Weight loss was highly variable and did not correlate with bleomycin dose (Fig. 1B). Furthermore, the development of pulmonary fibrosis in these mice varied significantly among experiments, both in severity and localization of lesions; in several regions, large areas of inflammation occur and show the acute nature of inflammatory response in the Hps1 model (Fig. 1C). While some mice showed almost no fibrosis histologically, others exhibited fibrosis that was more severe in central areas compared to the periphery (data not shown). The oropharyngeal administration route was found to be inefficient and inconsistent due to high mortality and variability in weight loss among the mice.

### Sensitivity of Hps1^*ep/ep*^ to subcutaneous administration of bleomycin

**Fig. 2:**
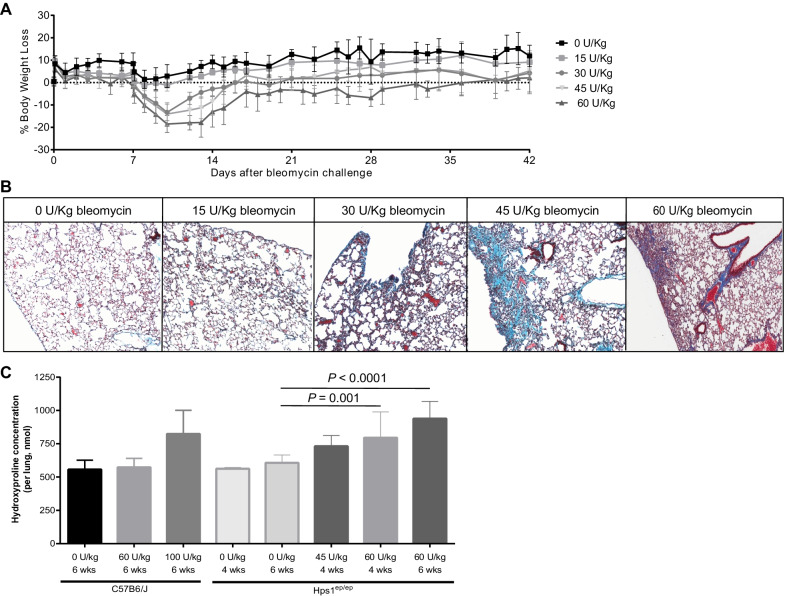
Effect of subcutaneous minipump administration of bleomycin in Hps1^*ep/ep* and^ C57BL/6 J wild type mice. **A** Percentage of body weight loss of Hps1^*ep/ep*^ mice after treatment with 0 to 60 U/Kg bleomycin compared to Day 0. Body weight loss appears to be dose-dependent. **B** Masson’s Trichrome stained lung slides (20×) of Hps1^*ep/ep*^ mice treated with 0, 15, 30, 45 and 60 U/Kg bleomycin. Fibrosis appeared dose-dependent. **C** Hydroxyproline (HYP) content of the left lung of both Hps1^*ep/ep*^ and C57BL/6J mice. Hps1^*ep/ep*^ mice exhibit higher susceptibility to develop bleomycin-induced PF. Statistical significance to determine *P* values as shown were ascertained through one-way ANOVA with multiple comparisons

Since the oropharyngeal administration of bleomycin led to a heterogenous fibrosis pattern, high mortality and a consequent survival bias, we modified the delivery of bleomycin and studied survival and development of pulmonary fibrosis. Delivery by subcutaneously implanted minipumps reduced the variation in bleomycin dosing, as these do not rely upon reflexive coughing in mice, but provide a slow and sustained bleomycin release into the subcutaneous tissue over the course of 7 days. Age- and weight-matched male Hps1^*ep/ep*^ mice were challenged with normal saline or bleomycin administered systemically via subcutaneous osmotic minipumps at doses of up to 60U/Kg; wild type (WT) C57BL/6J male mice received doses up to 100 U/Kg. We found that subcutaneous administration of saline or bleomycin at doses of 15, 30, 45 and 60 U/Kg in Hps1^*ep/ep*^ mice or 100 U/Kg in WT mice resulted in almost 0% mortality (data not shown). One Hps1^*ep/ep*^ mouse given 45U/Kg, however, died 5 days post minipump implementation, prior to pump removal, and it was likely due to complication to anesthesia; this mouse was excluded in the analysis as histopathology was not done. Body weight loss in Hps1^*ep/ep*^ mice was dose-dependent (Fig. 2A). Peak weight loss occurred 10–13 days post implantation of the osmotic minipump, and weight returned to baseline by 17–20 days post pump implantation. Lung histopathology demonstrated an increase in fibrotic lesions especially in the periphery of the lung and perivascular areas as the bleomycin doses increased from 0 to 60 U/Kg (Fig. 2B). Consistent with these data, lung hydroxyproline content increased in a dose-dependent manner at 4 weeks post pump implantation and in a time-dependent manner at a bleomycin dose of 60 U/Kg (Fig. 2B). Furthermore, hydroxyproline measurements indicated that 60 U/Kg of bleomycin induced severe fibrosis in Hps1^*ep/ep*^ mice but not in WT mice (Fig. 2C). Lung histopathology of WT mice that were challenged with 100 U/Kg exhibit smaller fibrotic lesions at different time points althouth the bleomycin dose they received was significantly higher; indicating the higher susceptibility of Hps1^*ep/ep*^ mice to bleomycin induce PF (Additional file 1: Fig. S1).

### Progressive pulmonary fibrosis induced by subcutaneous administration of bleomycin in Hps1^*ep/ep*^ mice

**Fig. 3:**
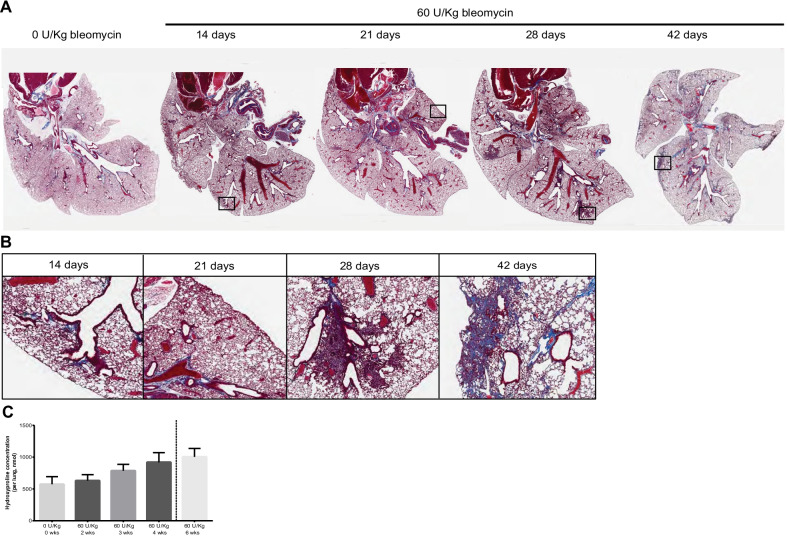
Hps1^*ep/ep*^ mice treated with 60 U/Kg bleomycin via subcutaneous minipump administration and euthanized 0, 2, 3, 4, and 6 weeks post pump implantation exhibited time-dependent fibrosis. **A** Masson’s trichrome stained lung slides (20×) of Hps1^*ep/ep*^ mice at different time points show more severe fibrosis over time. The fibrosis is shown more in the subpleural and the peribrochovascular areas of the lungs. **B** Magnified images in selected fibrotic areas that are shown in **A**. **C** Hydroxyproline (HYP) content of the left lung of Hps1^*ep/ep*^ mice as a fibrotic marker increases with time. One Way ANOVA test was done to calculate statistical significance

Hps1^*ep/ep*^ mice challenged with bleomycin delivered subcutaneously by osmotic minipumps exhibit a dose-dependent (Fig. 2B) and time-dependent (Fig. 3A–C) development of fibrosis as shown histologically and as measured by MS/MS quantification of hydroxyproline. As expected, Hps1^*ep/ep*^ mice presented higher susceptibility to develop bleomycin- induced PF compared to WT mice (Fig. 2C). Because Hps1^*ep/ep*^ mice developed severe fibrosis and had 100% survival after receiving 60 U/Kg of bleomycin via subcutaneous osmotic minipump, we performed additional experiments to study pulmonary fibrosis using this dose. Lung histopathology of subcutaneous bleomycin-challenged Hps1^*ep/ep*^ mice shows that fibrosis begins to develop 21 days post administration of bleomycin and increases in severity until day 42 (Fig. 3A, B). Focal regions of fibrosis were predominantly found in peribronchovascular areas and in subpleural regions; central lung areas contained relatively less fibrotic lesions. Masson’s trichome staining demonstrated densely stained collagen within fibrotic areas after day 28 (Fig. 3B). Hydroxyproline measurements in the lungs also exhibit time dependent increase that starts from day 21 and continuously until day 42 (Fig. 3C). Analysis of other organs harvested from subcutaneous bleomycin-challenged Hps1^*ep/ep*^ mice, including kidney tissue and skin tissue taken from regions distant from the suprascapular area, demonstrated no significant histological evidence of fibrosis at any timepoint (data not shown).

### Dynamics of inflammation and fibrotic development in subcutaneous bleomycin challenged Hps1^*ep/ep*^ mice

**Fig. 4:**
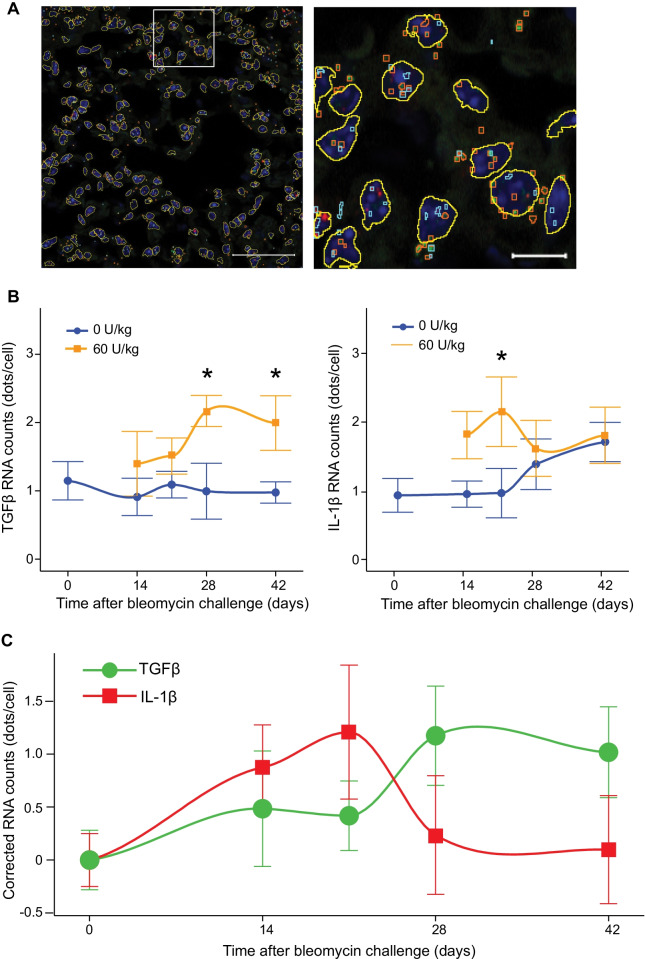
Quantification of inflammatory (IL-1β) and fibrotic (TGF-β) transcripts by RNAScope (Fluorescent in situ hybridization-based RNAScope assay). **A** Original (left) and magnified (right) images of bleomycin-challenged mouse lung tissue. RNAScope was used to detect TGF-β (green dots, mask outlined in orange) and IL-1β (red dots, mask outlined in turquoise) mRNA. Nuclei (blue, mask outlined in yellow) were stained with DAPI. Right panel shows magnified section of left image. ZenBlue 3.1 (Zeiss) software was used to generate masks to differentiate between true RNAScope signal and autofluorescence or background signal. Autofluorescence and background signal were excluded from the quantification of transcripts and nuclei. Size bar = 50 μm, left; 10 μm, right). **B** Quantification of transcripts of fibrosis (TGFβ) and inflammation (IL-1β) by RNAScope. Counts were adjusted for cell number as detected by DAPI nuclear count within the same image area. **C** Difference between the quantity of inflammatory (IL-1β) and fibrotic (TGFβ) markers between the mice challenged with 60 U/Kg bleomycin and age-matched controls as detected by RNAScope. Reported data display average of 10–13 images for each bleomycin dose and timepoint combination. Significance markers depict a difference with p < 0.01 for ANOVA followed by Tukey’s Post-Hoc Test

Bleomycin challenge in murine models of pulmonary fibrosis is associated with two distinct “phases” of fibrotic development, i.e. the inflammatory phase and the fibrotic phase [18]. To evaluate both inflammatory and fibrotic markers, RNA-fluorescence in situ hybridization (FISH) was performed on formalin-fixed, paraffin-embedded lung sections (Fig. 4A). Bleomycin-challenged Hps1^*ep/ep*^ exhibited a time-dependent upregulation of both the pro-inflammatory cytokine *Il1-β*, which reflects tissue injury and inflammation that accompanied disease initiation, and the pro-fibrotic mediator *TGF- β*, representing fibrogenesis in the lung (Fig. 4B). Levels of *TGF-β* initially remained near baseline after bleomycin challenge, only beginning to increase by day 28 post pump implantation and remain elevated through day 42. In contrast, levels of *Il1-β* were elevated at day 14, peaked at day 21, and returned to baseline at day 42 post pump implantation. These data demonstrate that the inflammatory phase preceded fibrogenesis in this model of pulmonary fibrosis induced by subcutaneous administration of bleomycin in Hps1^*ep/ep*^ mice (Fig. 4C). Additional signal quantification information is shown in Additional file 3: Table S1 and Additional file 2: Fig. S2.

## Discussion

Animal models mimicking human disease are needed to facilitate research focusing on understanding pathobiologic mechanisms, identifying therapeutic targets, and developing effective treatment. HPS-1 is characterized by the development of a highly prevalent pulmonary fibrosis in affected adults, and thus is a model for studying pulmonary fibrosis. Current murine models of HPS-1 do not develop spontaneous pulmonary fibrosis; previous studies, however, demonstrated that Hps1^*ep/ep*^ mice show increased sensitivity to lung fibrosis induced by intratracheal instillation of bleomycin [8, 12, 13, 19, 20]. Although insights have been made into the pathogenesis of HPS pulmonary fibrosis, studying this murine model of HPS pulmonary fibrosis has some limitations. We show in this paper that bleomycin challenge by oropharyngeal aspiration leads to inconsistensies in mortality, weight loss, and fibrotic development of challenged mice. Because this route relies on the aspiration reflex of mice to draw bleomycin into the lungs, it is likely that bleomycin distribution to different lobes of the lungs varies greatly depending on the strength of the aspiration response and the placement of the bleomycin solution into the trachea. The higher incidence of mortality after bleomycin challenge could be due to the severity of acute lung injury and inflammation, which are findings that do not recapitulate the natural history of human pulmonary fibrosis.

We report a method of bleomycin induced pulmonary fibrosis that was adapted from previous studies [17, 21, 22], and utilized this modified method in Hps1^*ep/ep*^ mice to more accurately model of HPS pulmonary fibrosis. We found that subcutaneous administration of bleomycin to Hps1^*ep/ep*^ mice resulted in sub-lethal weight loss, development of a discrete inflammatory response associated with expression of inflammatory markers, as well as biochemical, histological, and expression-based evidence for the gradual development of subpleural pulmonary fibrosis. This pattern differs from intratracheal bleomycin administration, which causes fibrotic changes that are most prominent around the airways [23]. Subcutaneous bleomycin administration leads to a delayed and distinct fibrotic phase after resolution of the inflammatory phenotype, which is consistent with models of idiopathic pulmonary fibrosis and scleroderma associated interstitial lung disease in which fibrosis was induced using subcutaneous minipump infusion of bleomycin [20]. Overall, these data are consistent with our findings in patients with HPS-1, where fibrosis has a very slow progression. In addition, we reported high concentrations of cytokines, chemokines, and alveolar macrophages in bronchoalveolar lavage fluid isolated from adult patients with HPS-1 who did not have radiographic evidence of pulmonary fibrosis [24]. Given the high penetrance of pulmonary fibrosis in adults with HPS-1, these findings indicate that lung inflammation precedes the development of pulmonary fibrosis in patients with HPS-1.

## Conclusion

In conclusion, we show that Hps1^*ep/ep*^ mice, when challenged with systemic bleomycin delivered via a subcutaneous administration route, slowly develop a progressive pulmonary fibrosis with characteristics that mimic human HPS pulmonary fibrosis. The development of inflammation and fibrosis in this model reliably occurs in two distinct phases, namely, inflammatory and fibrotic phases, facilitating the investigation of novel anti-inflammatory and anti-fibrotic therapeutics through discrete therapeutic windows. This model provides a low-mortality preclinical tool that will allow researchers to study the mechanism of pulmonary fibrosis in HPS, compare HPS pulmonary fibrosis to more common forms of fibrotic lung disease, and provide a platform for the development of therapeutics to treat HPS pulmonary fibrosis.

## Supplementary Information

**Additional file 1: Figure S1. **Lung histology of C57BL/6 J WT mice given 100U/Kg bleomycin via subcutaneous minipump. **(A)** Masson’s Trichrome staining lung slides (20X) of WT mice at different time points show more severe fibrosis over time. **(B)** Zoom in on selected fibrotic areas.

Additional file 2: **Figure S2**. RNA Scope mask parameters. Magnified images of blue (DAPI, left, 405 channel), green (TGF-β, center, 488 channel), and red (IL-1β, right, 555 channel) signals of bleomycin-challenged mouse lung tissue sections. Zen Blue 3.1 software was used to identify and quantify number of signals. Areas outlined in yellow, orange, and turquoise represent areas that were determined to be nuclei, TGF-β, or IL-1β, respectively. Areas outlined in white were determined to be background staining or autofluorescence and were excluded from quantification of nuclei and transcripts. Fluorescence in each channel is pseudo-colored in white to make fluorescent areas easily visible. Scale bar = 10 μm.

**Additional file 3: Table S1**: RNAScope Image Analysis Routine.

## Data Availability

All raw data of results described in the manuscript are available upon request.

## Abbreviations

HPS: Hermansky-Pudlak syndrome
HPS-1: Hermansky-Pudlak syndrome type 1
HPSPF: Hermansky-Pudlak syndrome with pulmonary fibrosis
PF: Pulmonary fibrosis
WT: Wild type

## Declarations

### Research involving animals

All experiments in mice were done accordance with NIH guidelines and approved by the NHGRI Animal Care and Use Committee (ACUC), under the protocol “Mouse models for disorders of lysosomes and lysosomal-related organelles”, with the approved proposal number, G-14-3.
