# Supplementary figures and images for: Progressive pulmonary fibrosis in a murine model of Hermansky-Pudlak syndrome
Source: Respir Res. 2022 May 4;23:112. doi: 10.1186/s12931-022-02002-z (PMC9066931; doi:10.1186/s12931-022-02002-z)

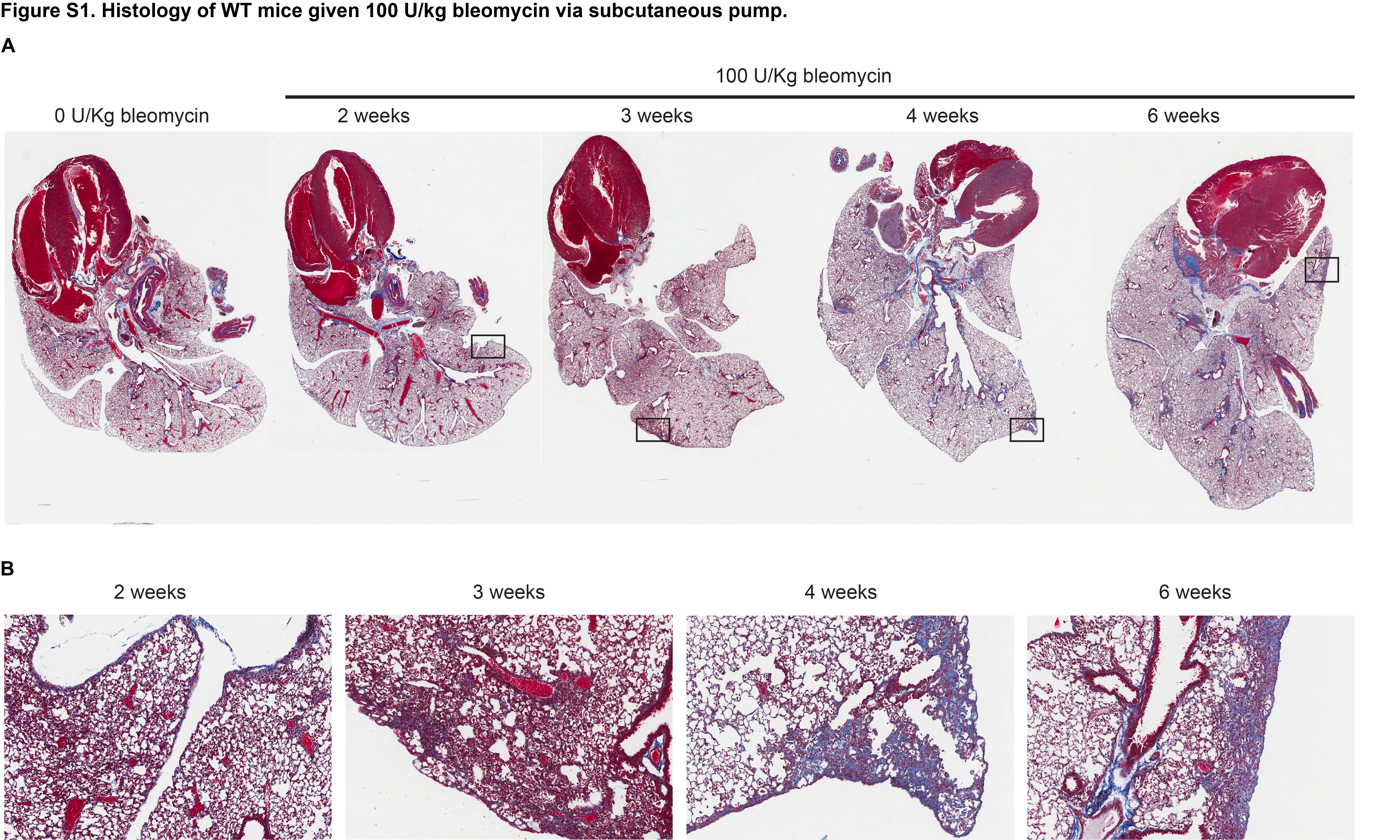

Supplement: Supplementary file 1 — Additional file 1: Figure S1. Lung histology of C57BL/6 J WT mice given 100U/Kg bleomycin via subcutaneous minipump. (A) Masson’s Trichrome staining lung slides (20X) of WT mice at different time points show more severe fibrosis over time. (B) Zoom in on selected fibrotic areas. [file 12931_2022_2002_MOESM1_ESM.png]

Figure S2

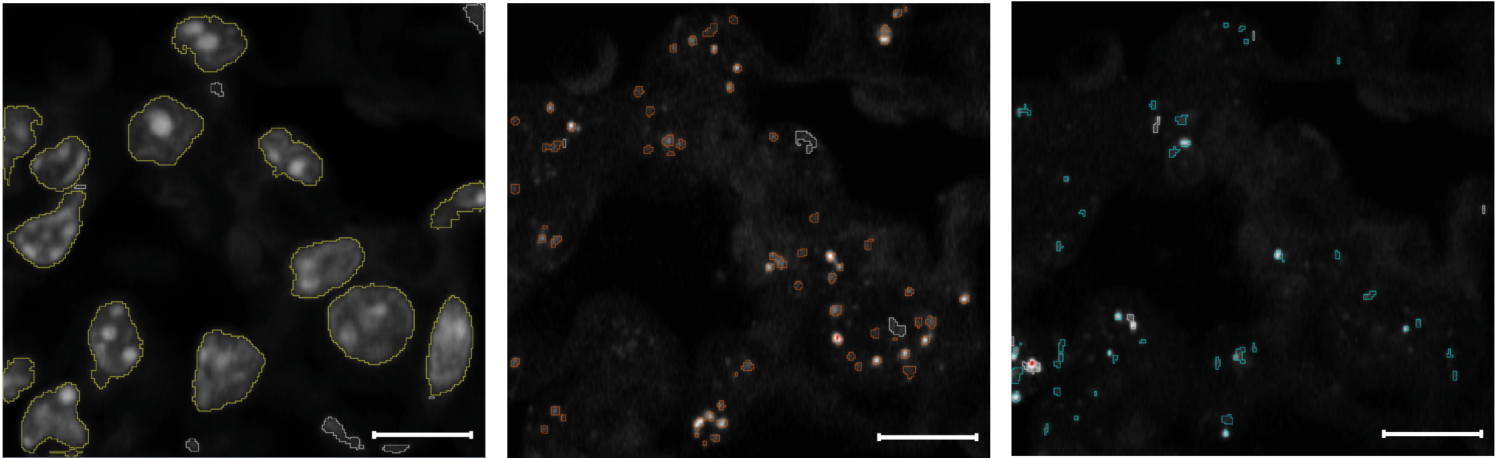

Supplement: Supplementary file 2 — Additional file 2: Figure S2. RNA Scope mask parameters. Magnified images of blue (DAPI, left, 405 channel), green (TGF-β, center, 488 channel), and red (IL-1β, right, 555 channel) signals of bleomycin-challenged mouse lung tissue sections. Zen Blue 3.1 software was used to identify and quantify number of signals. Areas outlined in yellow, orange, and turquoise represent areas that were determined to be nuclei, TGF-β, or IL-1β, respectively. Areas outlined in white were determined to be background staining or autofluorescence and were excluded from quantification of nuclei and transcripts. Fluorescence in each channel is pseudo-colored in white to make fluorescent areas easily visible. Scale bar = 10 μm. [file 12931_2022_2002_MOESM2_ESM.pdf]
