# Supplementary material for: Progressive pulmonary fibrosis in a murine model of Hermansky-Pudlak syndrome
Source: Respir Res. 2022 May 4;23:112. doi: 10.1186/s12931-022-02002-z (PMC9066931; doi:10.1186/s12931-022-02002-z)
Supplement: Supplementary file 3 — Additional file 3: Table S1: RNAScope Image Analysis Routine. [file 12931_2022_2002_MOESM3_ESM.docx]

**Table S1: RNAScope Image Analysis Routine**

|  | Nuclei  (DAPI, blue, 405 channel) | TGF-beta (fluorescein, green, 488 channel) | IL-1beta  (cyanine 3, red, 555 channel) |
| --- | --- | --- | --- |
| *Frame* | | | |
| Mode | Cut at Frame | | |
| *Automatic Segmentation* | | | |
| Segmentation mode | Segmentation with Background Subtraction | Segmentation with Background Subtraction | Segmentation with Background Subtraction |
| Smoothing | Lowpass, Size 5 | Lowpass, Size 3 | None |
| Background Subtraction | Rolling Ball, Radius 50 | Rolling Ball, Radius 8 | Rolling ball, Radius 5 |
| Sharpening | Unsharp Masking (Strength 5.0) | Delineate (Threshold 5, Size 3) | Delineate (Threshold 0, Size 1) |
| Thresholds | 7100-65535 (max) | 6900-65535 (max) | 6969-65535 (max) |
| Binary | None | None | Close, Count 1 |
| Separate | Watersheds, Count 4 | Watersheds, Count 9 | Watersheds, Count 2 |
| Tolerance | 1% | 3% | 3% |
| Neighborhood | 1 | | |
| Minimum Area | 1 | | |
| Min. Hole Area | 1 | | |
| Fill Holes | yes | | |
| Suppress Invalid | yes | no | no |
| *Region Filter* | | | |
| Circularity | 0.450-1.000 | 0.575-1.000 | 0.550-1.000 |
| Area | 10000000-max | 0-2000000 | 0-1500000 |
| Diameter |  | 200-1500 | 0.000-2000 |
| Compactness |  | 0.500-1.000 |  |
| Roundness |  | 0.300-1.000 |  |
